# Eye-Hand Coordination during Dynamic Visuomotor Rotations

**DOI:** 10.1371/journal.pone.0007004

**Published:** 2009-09-15

**Authors:** Lorenzo Masia, Maura Casadio, Giulio Sandini, Pietro Morasso

**Affiliations:** 1 Department of Robotics Brain and Cognitive Science, Italian Institute of Technology, Genoa, Italy; 2 Department of Informatics, Systems, Telecommunications, University of Genoa, Genoa, Italy; 3 National Institute of Neuroscience, Turin, Italy; The University of Western Ontario, Canada

## Abstract

**Background:**

for many technology-driven visuomotor tasks such as tele-surgery, human operators face situations in which the frames of reference for vision and action are misaligned and need to be compensated in order to perform the tasks with the necessary precision. The cognitive mechanisms for the selection of appropriate frames of reference are still not fully understood. This study investigated the effect of changing visual and kinesthetic frames of reference during wrist pointing, simulating activities typical for tele-operations.

**Methods:**

using a robotic manipulandum, subjects had to perform center-out pointing movements to visual targets presented on a computer screen, by coordinating wrist flexion/extension with abduction/adduction. We compared movements in which the frames of reference were aligned (*unperturbed condition*) with movements performed under different combinations of visual/kinesthetic dynamic perturbations. The visual frame of reference was centered to the computer screen, while the kinesthetic frame was centered around the wrist joint. Both frames changed their orientation dynamically (angular velocity = 36°/s) with respect to the head-centered frame of reference (the eyes). Perturbations were either *unimodal* (visual or kinesthetic), or *bimodal* (visual+kinesthetic). As expected, pointing performance was best in the unperturbed condition. The spatial pointing error dramatically worsened during both unimodal and most bimodal conditions. However, in the bimodal condition, in which both disturbances were in phase, adaptation was very fast and kinematic performance indicators approached the values of the unperturbed condition.

**Conclusions:**

this result suggests that subjects learned to exploit an “affordance” made available by the invariant phase relation between the visual and kinesthetic frames. It seems that after detecting such invariance, subjects used the kinesthetic input as an informative signal rather than a disturbance, in order to compensate the visual rotation without going through the lengthy process of building an internal adaptation model. Practical implications are discussed as regards the design of advanced, high-performance man-machine interfaces.

## Introduction

When performing a visuomotor task it is necessary to integrate visual and kinesthetic information that may be spatially dissociated. Looking at a mirrored image implicates a spatial dissociation between the visual and proprioceptive information [1]–[2], and what we see is an artificial representation of what we touch. The previous example comprises a multitude of common situations of sensory dissociations and visuomotor distortions; technology developments have provided new aiming tools to be used in an unusual sensory environment, implementing a novel visuomotor transformation integrating vision and proprioception.

For example using a computer mouse requires to associate the hand movements on a table with the cursor movements on a screen; apparently this kind of transformation is readily mastered by any subject, without the need of a time-consuming training. Other examples are teleoperation or, telesurgery which are known to be particularly demanding due to their accuracy and precision requirements [3]–[5]. In these situations the nervous system is forced to associate spatially separated signals and unify their percepts to obtain a coherent interpretation and providing the right motor command [6]–[7]. The rotational misalignments (visuo-motor rotation) between a coordinate system associated with the visual scene, typically obtained through a remote sensor, and the motor coordinates under the operator's control, are mentally challenging and tiresome, and only experience decreases the required time especially in those tasks where execution needs to be accurate and prompt. Besides compensating a visual rotation is a much more burdensome transformation than scaling or translation; even in the restricted case of a rigid transformation in the frontal plane, equivalent to the retinal plane, rotations are more challenging than translations to the machinery of shape interpretation and recognition, because readily extractable visual features, as vertical and horizontal lines, remain invariant under translation but not under rotation.

Visuo-motor rotations have been investigated by using two main types of simplified experimental paradigms in order to evaluate position perception and movement production under visuo-proprioceptive discrepancies: first, introducing a visual bias by optical prisms or virtual reality (VR) to asses accuracy of target reaching [8]–[10]; second, by using step-wise position/rotation offsets or vibratory stimulation to create proprioceptive disturbances of the limb [11]–[19]. One observed that when a distortion of this kind is introduced repeatedly with different amplitudes, the subjects' visuomotor performance is initially disrupted but gradually normalizes over the course of a prolonged exposure [20]–[26]: these outcomes fit well with the view that adaptation is achieved by a gradual modification of an internal reference frame [27]–[28]. With static orientation perturbation of the visual scene, motor errors were found to differ as a function of the perturbation magnitude and appear to be maximal at 90 degrees [29]–[33], which represents a limit case for adaptation. However static visuomotor rotation is rarely seen in typical remote control or telemanipulation applications. Therefore, this work aims to investigate human performance in a dynamic visual distortion using multisensory integration by means of a kinesthetic cue; in contrast with previous studies the visual disturbance will be coupled to a kinesthetic one, and their mutual orientation will be time-varying and not static or stepwise; a wrist pointing task will be used instead of the more common arm reaching paradigm for two main reasons: first, the anatomy of the wrist allows to use one of the three degrees of freedom as an input channel for a kinesthetic perturbation; second, wrist is the most involved joint when interacting with human-computer interfaces.

Can we expect, as in most studies reported in the literature, that in response to a visual or kinesthetic perturbation, presented separately, adaptation is lengthy and requires building an appropriate internal model of the perturbation? The situation is even less clear if both perturbations are applied at the same time, thus inducing a “redundant” and “bimodal” disturbance pattern, with the freedom to modulate the phase shift between the two components (visual and proprioceptive). It is possible indeed that, in specific experimental conditions, an invariant relationship among the two perturbations can emerge as an “affordance” to be detected by the subjects as a kind of shortcut to be exploited in a quick manner without the need to build the internal model of compensation. If this is the case, we may exploit this effect for the design of human-computer interfaces that allow fast adaptation in a number of remote-control tasks.

Recent visuomotor adaptation studies revealed indeed how quick is the visuomotor system at building associations that can simplify or reduce the computational work-load. For example, evidence has been found about the transfer of adaptation between ocular saccades and arm movements [34]; the underlying neural correlates of adaptations have been studied by Girgenrath et al [35]. The effect of aging on such adaptive abilities has also been investigated [36].

The neural correlates of wrist pointing movements have been the subject of several monkey studies [37], [38], [39] that addressed the fundamental processes that transform sensory signals to generate a goal-directed movement. Insight into this process of sensorimotor transformation was obtained by examining the coordinate frames of neuronal activity in interconnected regions of the brain. The activity of neurons in primary motor cortex (M1) and ventral premotor cortex (PMv) was recorded in monkeys trained to perform a task which dissociates three major coordinate frames of wrist movement: muscle, wrist joint, and an extrinsic coordinate frame. Three major types were found in both cortical areas: 1) ‘extrinsic-like’ neurons, whose activity appear to encode the direction of movement in space, independent of the patterns of wrist muscle activity or joint movement that produced the movements; 2) ‘extrinsic-like neurons with gain modulation’, whose activity encodes the direction of movement in space, but the magnitude (gain) of neuronal activity depended on the posture of the forearm; 3) ‘muscle-like’ neurons, whose activity co-varied with muscle activity. These results support the hypothesis that rather abstract information like spatio-temporal patterns in extrinsic coordinates are indeed represented in the cortex and raise the possibility that cortical processing between M1 and PMv may contribute to a sensorimotor transformation between extrinsic and intrinsic coordinate frames. This is the necessary neural substrate for carrying out the visuo-motor tasks investigated in this study.

In this context, the open questions addressed by this paper are the following: “is visual feedback predominant with respect to proprioception or can subjects develop a visual-proprioceptive synergy of visuomotor coordination in order to accomplish the pointing task in dynamic conditions?” Moreover, “does the synergy emerge through a slow adaptation process or is it readily available to the brain machinery?”

## Methods

### 2.1 Subjects

The research was approved by the Italian Institute of Technology Review Board and conforms to the ethical standards laid down in the 1964 Declaration of Helsinki, which protects research subjects. Before beginning each subject signed a consent form that conforms to these guidelines.

Eighteen unimpaired male subjects (age: 25±1.3 y) with no history of neurological disease participated to the experiments. They were all right-handed and naïve to the experimental setup. The subjects were randomly assigned to three age-matched groups, with the same number of participants, in order to evaluate the effect of stimulation sequence on their performance.

### 2.2 Experimental Set up

**Figure 1:**
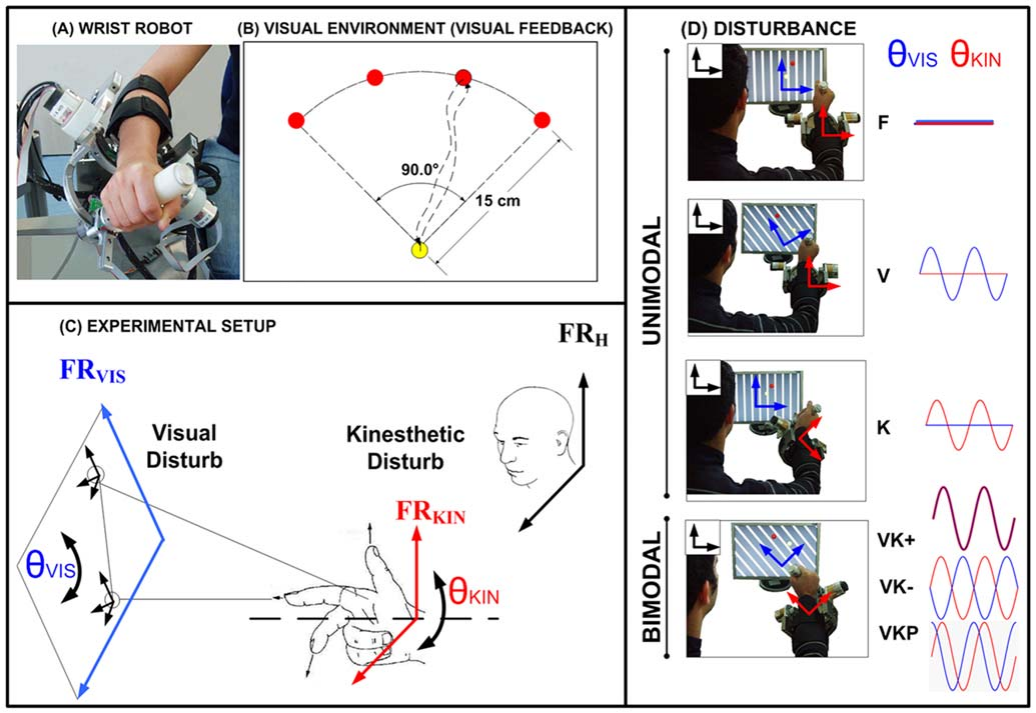
Apparatus and experimental procedures. (A): Wrist robot (WR) and (B): Visual Environment (VE) task is to perform center-out pointing movements, using the F/E and Ab/Ad DOFs, to each one of the four different targets: a central target, which corresponds to the neutral wrist position (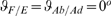) and four peripheral targets equally spaced on the upper semi-circle. (C):the experimental protocol the *x* and *y* coordinates of VR (FR_VIS_) always correspond to movements of the F/E (flexion/extension) and Ab/Ad (abduction/adduction) wrist DOFs (FR_KIN_) respectively. (D): unimodal and bimodal disturbances: F condition; K condition; V condition; VK+ or VK− or VKP condition. The red circle identifies the target and the yellow circle the wrist end effector. The orientation of the visual scene is identified by the stripe pattern. In the case of kinesthetic disturbance K the P/S DOF was driven by a position servo with no effect on the VR. In the case of the visual disturbance V, the visual scene was rotated with respect to the computer screen. Bimodal conditions are a combination of both visual and kinesthetic disturbances. The VK- and VKP conditions are similar bimodal perturbations as VK+ but with a variable misorientation of the visual frame FR_VIS_ and the wrist frame FR_KIN_.

The device used in the experiments is a Wrist robot (Figure 1.A) which was developed for motor control studies and rehabilitation. It has 3 DOFs (Degree of Freedom): F/E (Flexion/Extension); Ab/Ad (Abduction/Adduction); P/S (Pronation/Supination). The corresponding rotation axes meet at a single point. It allows the following range of motion (ROM): 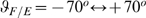; 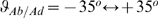; 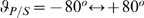. These values approximately match the ROM of a typical human subject. The subjects held a handle connected to the robot and their forearms were strapped to a rigid holder in such a way that the biomechanical rotation axes were as close as possible to the robot ones. Unavoidable small misalignments were compensated for by means of a sliding connection between the handle and the robot.

The control architecture of the task integrates a) the wrist controller with b) a bi-dimensional visual environment (VE). The F/E DOF corresponds to the *x* (horizontal) axis of the VE and the Ab/Ad DOF t of the *y* (vertical) axis.

The wrist controller leaves the Ad/Ab and F/E DOFs un-actuated, whereas it implements a high-stiffness control scheme on the P/S DOF with two alternated operating modes during the different phases the experimental protocol: 1) maintaining the initial neutral P/S angle; 2) introducing a proprioceptive perturbation by enforcing a sinusoidal oscillation of the P/S indicated as θkin.

VE shows to the subjects on a computer screen the actual pointing direction of the hand (as a sort of virtual hand-held laser pointer) and the corresponding target direction, both represented as round circles of different colors against a textured background. The pointing direction is fed back on the computer screen using the Ad/Ab and F/E angular readouts with an appropriate scale factor (1 rad = 0.25 m); the P/S readout is not used for the pointing task. The VE software can also carry out a function of visual perturbation, by superimposing a sinusoidal rotation on the displayed patterns, including the background.

### 2.3 Experimental Protocol

The experimental protocol was designed in order to explore the sensorimotor transformations, from a representation of target position to the intended movement in the context of a pointing task. A crucial point is to identify the coordinate frames in which these motor computations are carried out. Assuming that the hand reference frame is coherent with the well known “right hand rule” (a Euclidean orthonormal frame made by index, medium fingers, and thumb of the right hand), it is possible to define a pointing task in the following manner: the combination of angular rotations in wrist spherical coordinates which align the index-vector with the target position in the space. A wrist pointing task involves multimodal sensory information for the computation of angular rotations: the position of the target in the world is remapped in retinal frame and the motor command are planned visually and mapped to an internal representation of movement in the world; finally the planned movement is mapped to a reference movement for the wrist and input to the wrist control system; therefore, a crucial element of the task is to express the different sensory signals from vision and proprioception into a common coordinate frame.

The task is to perform center-out two-dimensional pointing movements, using the F/E and Ab/Ad DOFs, to each one of four different targets: a central target, which corresponds to the neutral wrist position (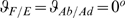) and four peripheral targets equally spaced on a semi-circle (Figure 1.B). The experiments are organized in blocks of trials, each one of them consisting of 10 target-sets. Therefore, each block includes 40 center-out movements and 40 return movements.

The task is defined in relation with three reference frames, which are presumably used by the central nervous system in order to control visually guided reaching/pointing movements in dynamic conditions [40]:

- A *head-centered* (or body-centered or ego-centric) frame of reference FR_H_;
- A *kinesthetic-wrist-centered* frame FR_KIN_;
- A *visual environment* allo-centric frame of reference FR_VIS_, which identifies the visual scene and the corresponding visual targets.

The screen is positioned in front of the subject in such a way that the forearm direction is approximately perpendicular to it (Figure 1.C). The orientation of the FR_VIS_ (θvis) is displayed by means of a stripe-shaped background that appears vertical on the computer screen unless a visual perturbation is operational.

The experimental protocol was performed in six different conditions (Figure 1.D):

- **F**: neutral or familiarization condition, intended to allow the subjects to adapt to the robot kinematics and dynamics and achieve a consistent accuracy. VE generates the targets and displays, with a circle of equal radius, the instantaneous orientation of the wrist. The robot control maintains the P/S DOF in the reference angular position and leaves the other DOFs inactivated.
- **K**: kinaesthetic perturbation. VE is the same as in the F condition. The robot control applies to the P/S DOF a disturbance by means of an harmonic oscillation with a frequency *f* = 0.1 Hz and an amplitude 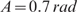 imposed to the *kinesthetic-wrist-centered* frame FR_KIN_ by means of a rotation angle θkin. In this condition the subjects has to re-compute the motor control commands in order to take into account the disturbance introduced by the imposed prono/supination of the wrist.
- **V**: visual perturbation. In this condition VE is modified by introducing a harmonic rotation of the visual scene FR_VIS_ (θvis) with the same frequency and amplitude of the previous **K** condition. In other words, the stripe pattern displayed on the screen is rotated together with the target and the circle representing the wrist orientation. Robot control is the same as in condition **F** holding the neutral P/S anatomical position. Also in this case the subjects has to re-map the motor control commands in order to take into account the disturbance applied to the visual input.
- **VK+**: this is the combination of the **V** and **K** conditions. Both disturbance inputs (visual and kinaesthetic) are applied at the same time, with the same frequency, amplitude and phase.
- **VK−**: this condition is similar to **VK+** with the difference that the two disturbance inputs rotate in opposition (phase lag = 180°).
- **VKP**: in this condition the two disturbance inputs are rotating with a phase lag of 90°, the **K** disturbance leading the **V** disturbance.

It may be observed that it is not clear to which extent the described experimental paradigm models conditions likely to be encountered in real life, as in telemanipulation or minimally invasive surgery. However, we may observe that a typical problem is to use a joystick or similar input device for reaching a target whose frame of reference rotates with respect to the user in a smooth way. This corresponds quite well to the condition **V** of the protocol. The other conditions were added to the protocol in order to understand the specific roles of visual and proprioceptive information in this visuomotor task (watch Audio/Video file Movie S1 for a better comprehension of the different experimental conditions).

For the sake of clarity, let us also define mathematically the mapping between wrist and cursor motion in the various experimental conditions. This mapping is defined by the following equation, which transforms the position of the cursor in the frame FR_VIS_ (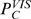) into the corresponding position in the frame FR_KIN_ (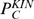):
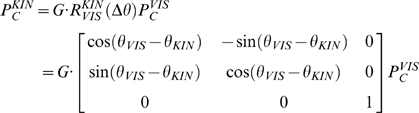




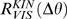
 is the rotation matrix from frame FR_VIS_ to FR_KIN_ and **G** is the scale factor for converting radians into pixels. It is worth noting that target-switching and the disturbance generation process are asynchronous, therefore in the experimental conditions **VK−** and **VKP** the angular difference 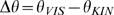 between the visual and the kinaesthetic patterns at the moment of target presentation (visuo-kinaesthetic misalignment) can have any value in the range of oscillation disturbance (±*A*). On the contrary, in the **VK+** condition the angular difference is always null because to the synchronous rotation of the two frames and the mapping will consist only in a multiplication of the joint motion of the wrist for the scale factor **G**.

The experimental protocol, summarized in table 1, is defined as follows:

- The experiments last 5 days for all groups, with 3 blocks of trials for each day.
- Each block includes 10 target-sets (40 center-out+40 return movements) and is identified by one of the 6 experimental conditions.
- The first block of each day is an **F** block, in order to allow the subjects to acquire an initial stable state (F1 to F5). The other two blocks are characterized by different combinations of experimental conditions as listed in the table.

**Table 1:** Sequence of experimental conditions for each day and each group.

Subjects/Day	Day-1	Day-2	Day-3	Day-4	Day-5
Group 1	F1 V1 V2	F2 K1 K2	F3 VK1+ VK2+	F4 VK1− VK2−	F5 VK1P VK2P
Group 2	F1 K1 K2	F2 V1 V2	F3 VK1+ VK2+	F4 VK1− VK2−	F5 VK1P VK2P
Group 3	F1 VK1+VK2+	F2 V1 V2	F3 K1 K2	F4 VK1− VK2−	F5 VK1P VK2P

F/V/K/VK+/VK-/VKP refer to the 6 experimental conditions. The numerals refers to number of times a group has been exposed to a given condition (e.g. VK2- means the second time the group received the VK- condition).

The purpose of this procedure was to verify whether or not there is a sequence effect in the response of the subject to different combinations of visual-kinesthetic perturbation.

### 2.4 Analysis

The two components of the pointing trajectories, i.e. the angular values of the F/E and Ab/Ad DOFs, were sampled at 100 Hz and smoothed by using a 6th order Savitzky-Golay filter, with a 170 ms window (cut-off frequency: ∼11 Hz). The same filter was also used to estimate time derivatives of the trajectory. From such data assuming that *Movement onset* is evaluated by detecting when the pointing speed exceeds a threshold of 0.1 rad/s while *Movement termination* is evaluated by detecting when speed falls below the same threshold, we estimated the following indicators:

- *Movement Duration*: time difference between movement onset and movement termination;
- *Average speed*: it is the mean value of the wrist angular rotation from movement onset to termination;
- *Aiming error*: it is the angular deviation from ideal trajectory (the straight line that connects the starting point to the target), evaluated 300 ms after movement onset;
- *Lateral deviation*: it is the maximum value of the distance between the pointing trajectory and the ideal trajectory, calculated between onset and termination times. It is a measure of the path curvature;
- *Jerk index*: it measures the smoothness of the trajectory and is calculated from the trajectory jerk 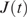 (norm of the third time derivative of the trajectory), by computing the square root of the averaged norm of *J*, normalized with respect to duration *T* and path length *L*: 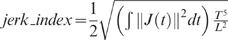
_._

Differences across conditions and groups were assessed by two factor ANOVAs for repeated measures; a contrast analysis (significance level *p* = 0.05) was used to rank the block effects on the pointing performance. A further statistical analysis (multivariate ANOVA for parallelism test) was also used to compare the goodness of fit of the aiming error as a function of the instantaneous rotational misalignment of the visual and kinesthetic frames under different conditions, as explained in the next paragraph.

## Results

### The experiment showed high similarities among all the participating subjects

**Figure 2:**
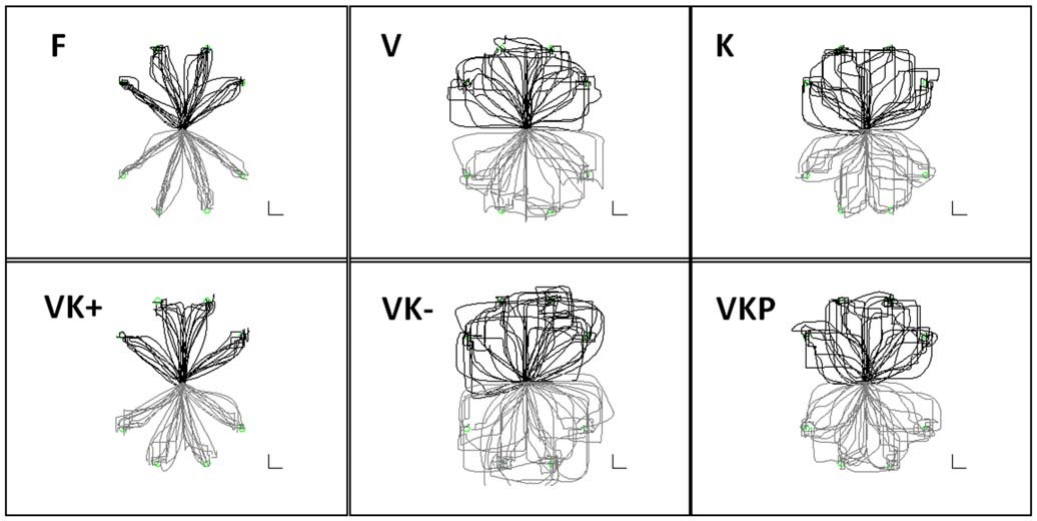
Pointing trajectories. For one of the subjects, the figure shows pointing trajectories in the 6 experimental conditions (F, V, K, VK+, VK−, VKP). Black trajectories correspond to center-out movements. Grey trajectories, which are displayed in a mirror way for graphical clarity sake correspond to return movements. Abscissas: F/E rotations or movements along x-axes; Ordinates: Ab/Ad rotations or movement along y-axes. The scale bars correspond to 2.5 cm on the computer screen or 0.1 rad in terms of wrist rotation.

In condition **F**, all the subjects exhibited pointing movements that are approximately straight in the F/E-Ab/Ad plane (see panel F in figure. 2) and with a bell-shaped speed profile. This suggests us that the underlying motor control mechanisms for arm reaching and wrist pointing are similar, in spite of the fact that they use different DOFs of the wrist. Moreover, it makes us confident that the intrinsic mechanical impedance of the robot did not alter the kinematics of the recorded movements. The same figure panel also shows that center-out movements and return movements have strong resemblance, although the latter ones display a lower degree of variability that can be explained by the lower uncertainty on the control parameters.

In the other experimental conditions, characterized by different combinations of visual-proprioceptive disturbances (unimodal and bimodal), it appears, from a qualitative observation of the plots, that the subjects are still able to reach the targets but trajectories in the kinesthetic frame of reference are markedly different in most cases. This is hardly surprising because pointing while compensating concurrent disturbances is clearly more complex than simple pointing and we may expect a significant modification of the control patterns required for reaching the targets. What is astonishing is that the effect on movement curvature is smaller if both disturbances are presented (panel **VK+** in fig. 2) than in the unimodal contitions, either visual or proprioceptive (panels **V** and **K**, respectively). However, this counterintuitive effect is present only if the two disturbances are in phase (compare panels **VK−** and **VKP** with **VK+** in fig. 2). The following detailed kinematic analysis clarifies and quantifies such qualitative initial observations.

One should also consider that in the conditions **F**, **VK+** the visuo-motor mapping between FR_KIN_ and FR_VIS_ remains fixed during the actual movement because 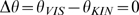; therefore the trajectories in the kinesthetic frame of reference (F/E, Ab/Ad) and the screen frame of reference (X/Y) are the same and they are equally oriented respect to head-centered frame FR_H_.

Contrarily in the other conditions (**K**, **V**, **VK−**, **VKP**) the visuo-motor mapping changes dynamically and thus the trajectories in the two frames of references FR_KIN_ and FR_VIS_ have same shapes but they are differently oriented respect to FR_H_. If one should consider to plot the trajectories in the head-centerd frame FR_H_ it will result impossible to display them on a static figure, because target switching is asynchronous with respect to the oscillation of the visual and kinesthetic frames.

**Figure 3:**
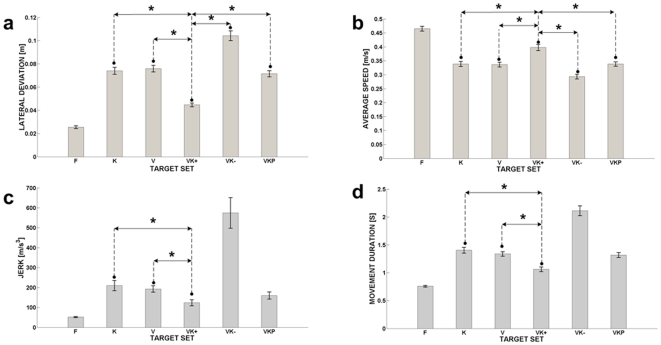
Results of kinematic analysis. a: lateral deviation of the pointing movements as a function of the different movement sets or experimental conditions. b: mean speed of the pointing movements (deg/s) as a function of the different movement sets or experimental conditions. c: Jerk index (rad/s^3^) of the pointing movements as a function of the different movement sets or experimental conditions. d: duration time (s) of the pointing movements as a function of the different movement sets or experimental conditions. *p<0.05 indicates a significant difference.

Figure 3.a, which displays the lateral deviation of the pointing movements from the ideal trajectory in the different blocks of trials, allows us to point out a number of relevant aspects, confirmed by the statistical analysis:

- The performance in the **F** conditions is quite uniform across the groups of subjects and the different experimental days: this confirms that the robot was not “invasive” and the subjects had no difficulty to adapt to it;
- The deviation is markedly higher in all the perturbed conditions in comparison with the **F** condition, as expected (F(5,75) = 309.2; p<0.01);
- The deviation in the **VK+** condition is significantly smaller that in all the other perturbed conditions; a contrast analysis shows significant differences between bimodal and unimodal conditions VK+, V and VK+, K (F(1,15) = 147.64; p<0.01; F(1,15) = 306.63; p<0.01, respectively).
- There is no significant group effect, i.e. the performance in the different conditions does not depend upon the sequence according to which the different conditions were experienced (F(2,15) = 3.521; p = 0.15).

The analysis of the average speed (Figure 3.b) shows a complementary behavior with respect to the lateral deviation, shown in figure 3.a: the speed is lower in the perturbed (**K**, **V**, **VK+**, **VK−**, **VKP**) than in the unperturbed trials (**F**) but the effect is quite smaller in the **VK+** condition (F(5,75) = 49.09; p<0.01). This finding confirms that adding the kinesthetic perturbation in phase with the visual perturbation has a facilitating effect on the pointing performance. The jerk index (Figure 3.c) shows that, as expected, while no significant differences was found among the groups (F(2,15) = 0.1063; p = 0.89), the movements in the F condition were strongly smoother than in all the perturbed conditions and a strongly significant difference was found among the different target sets (F(5,75) = 60; p<0.01). On the other hand, the comparison among the different disturbed conditions shows an equivalent degree of smoothness with the exception of the VK− situation that appears to be more affected than all the others.

Movement duration is often used in experimental psychology to measure the duration of mental operations and as indicator of task complexity. Indeed duration is a measure of information processing and it is an external indicator of the ability of the nervous system to receive, process, initiate and complete a response to incoming stimuli. Movement that take more time to be performed are assumed to require longer information processing times, and thus are considered to be more complex for the central nervous system. Figure 3.d shows a significant reduction in movement duration for VK+ condition respect to the other unimodal (V and K) and bimodal (VK− and VKP) target sets.

**Figure 4:**
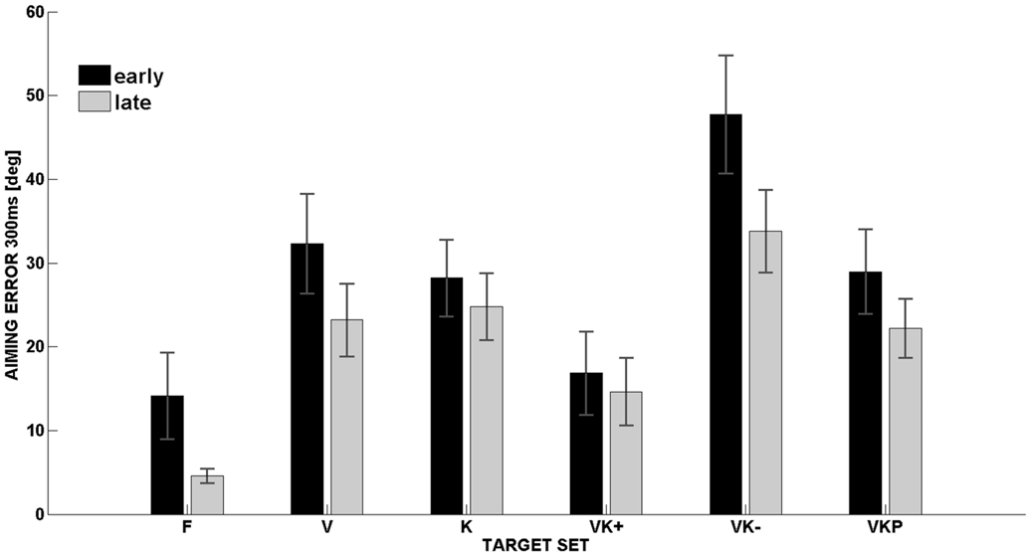
Aiming error adaptation. Aiming error (deg) of the pointing movements, at 300 ms after movement onset or experimental conditions, as a function of the different movement sets. The aiming error was evaluated at the first 15 trials and last 15 trials to see if an adaptation occurs during the different target sets.

The plot of the aiming error at 300 ms after movement onset (Figure 4) gives a similar picture to the one offered by the lateral deviation (Figure 3.a); no significant differences among the groups (F(2,15) = 0.208; p = 0.814) were observed and highly significant differences between the target sets (F(5,75) = 231.84; p<0.01). The aiming error was evaluated for the first 15 and last 15 trials; as shown in the figure subjects tend to improve their performance in both unimodal and bimodal conditions and adapt to the new visuomotor transformation, even if the rotational misalignment is never constant but continuously varies during the task. Although an adaptation occurs, it is noticeable that the **VK+** condition presents significant reduction in terms of aiming error if compared with both unimodal and bimodal target sets.

Moreover, the aiming error also allows a different kind of analysis that may shed light on the mechanism according to which performance depends on experimental conditions. The idea is to correlate the aiming error, calculated 300 ms after movement onset, with the angular difference between the orientation of the visual scene (θvis, generated by the VE module rotating the extrinsic visual reference frame FR_VIS_) and the orientation of the P/S DOF (θkin, generated by the robot controller which rotates the intrinsic wrist reference frame FR_KIN_ ), measured at the same time instant. This angular difference or visuo-kinaesthetic misalignment is zero in the **F** condition because both angles are fixed; it is variable in the **V** and **K** conditions because one of the angle remains fixed but the other is varying; it is also variable in the **VK−** and **VKP** conditions because both angles vary as well as their difference; however, the angular difference is persistently null in the **VK+** condition because in this case both the visual and proprioceptive angles oscillate but remain perfectly in phase. The point, as already noted in the methods, is that the target selection process is asynchronous with respect to the disturbance generation process and this means that in the conditions in which the visuo-kinaesthetic misalignment is time-varying (**V**, **K**, **VK−**, **VKP**) the value of such angular difference when a pointing movement is initiated is randomly distributed, with a distribution that is approximately uniform in the possible range of values.

**Figure 5:**
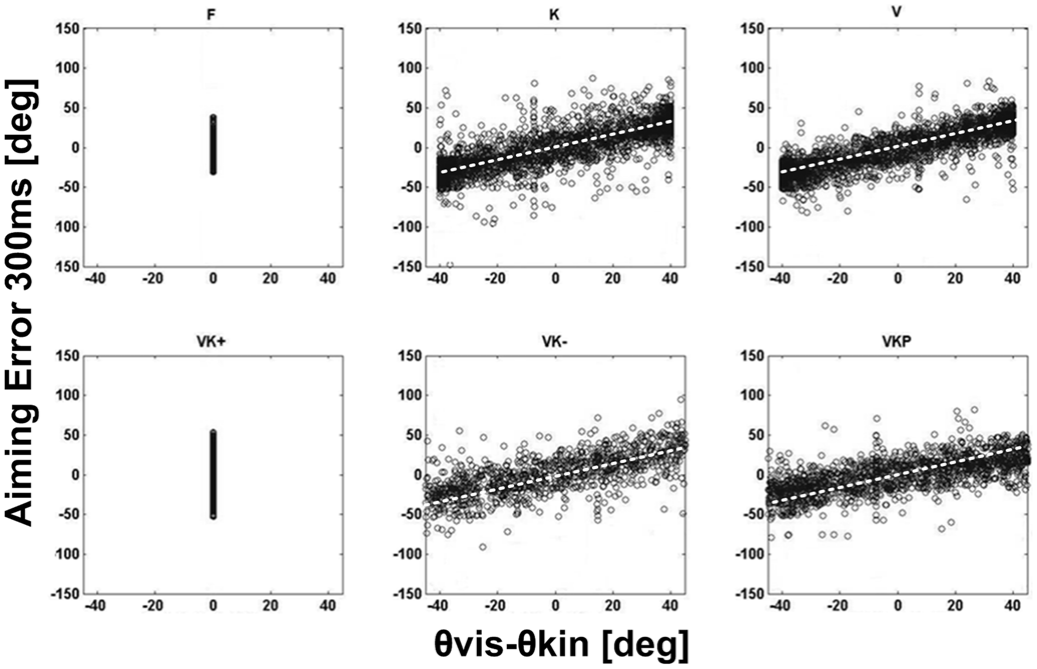
Aiming error as function of instantaneous visuo-kinesthetic rotational misalignment. Scatter diagram, for the whole population of subjects, of the aiming error 300 ms after movement onset as a function of the rotational misalignment between the visual disturbance (θvis) and the kinaesthetic disturbance (θkin). The misalignment is null by definition in the F and VK+ conditions; it is randomly distributed across the whole range of possible values in all the other conditions. The slopes of the regression lines have the following values, for the K, V, VK− and VKP conditions, respectively: 0.749, 0.799, 0,697, 0.679; these values all differ significantly from 1.

Figure 5 shows the scatter diagram of the aiming error at 300 ms as a function of the corresponding angular difference (θvis-θkin) or visuo-proprioceptive misalignment for the three groups (a total of 2720 points for each plot). We pooled the data from the whole population of subjects because the statistical analysis of the aiming error did not exhibit any statistical difference among the three groups of subjects. For the **F** and **VK+** conditions, in which the visuo-proprioceptive misalignment is null, the figure shows the variability of the aiming error, which is higher in the **VK+** than in the **F** situation (standard deviation: 28° vs. 18°), as could be expected. In the other situations the figure shows that visuo-proprioceptive angular misalignments occur in the whole range of possible values and are approximately distributed in a uniform way. The figure also suggests a linear trend in the relationship between the aiming error and the misalignment that appears to be similar in all the conditions. This is also confirmed by the regression analysis of the scatter diagrams, evaluated at 95% confidence level. The R-square values in the different experimental conditions were remarkably high: 0.837 (**K**); 0.857 (**V**); 0.868 (**VK−**); 0.777 (**VKP**). The slopes of the four regression lines were compared using a Profile analysis (application of multivariate analysis) and they did not exhibit significant statistical differences (F(3,1876) = 0.323; p = 0.8089).

When the two disturbances are simultaneously applied to the two channels (FR_VIS_ and FR_KIN_) (**VK+** condition), one could expect an additive deterioration of the pointing performance. However, what happens is just the opposite: the pointing accuracy is almost as good as in the unperturbed condition (**F**). One can observe that in such condition, although the two channels are both disturbed, the visuo-proprioceptive misalignment remains null at any time, i.e. the two frames of reference coincide although both rotate with respect to the environment frame.

In order to put the misalignment conjecture on more solid bases, we designed a bimodal perturbation experimental paradigm with a time-varying visuo-proprioceptive misalignment (**VKP**, **VK−**): it turns out that the aiming error is at its minimum value when the misalignment angle is null and the error grows in a linear way in relation with the misalignment, as shown in fig. 5. The reasons for this kind of phenomenon can be explained by observing the rotation of the two reference frames FR_VIS_ and FR_KIN_ during each of the different experimental conditions.

**Figure 6:**
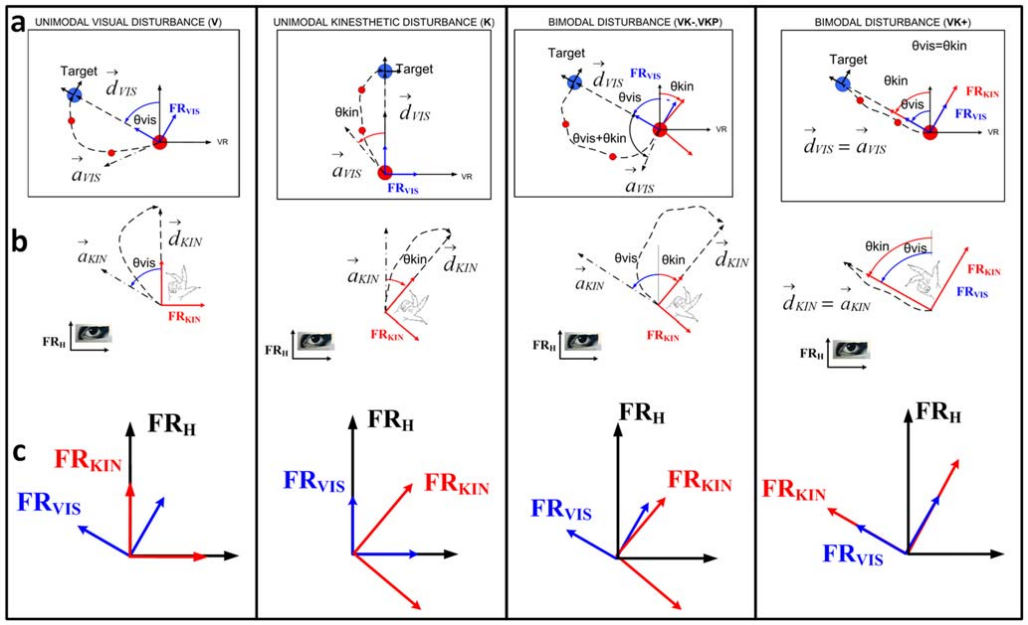
Instantaneous mutual orientation of kinaesthetic and visual frames and trajectory generation. (a): screen visualisation of the intended movement towards the target (blue circle) using the cursor (red circle); (b): wrist movement in order to reach the target in different experimental conditions. The curved path wrist movement are mapped on the screen rotated according the visuomotor transformation. The inability of the subject to move the wrist along the desired direction d_kin_, which would correspond to the straight path to the target d_vis_, is caused by the lack of capacity in mentally rotating the kinaesthetic-wrist-centered FR_KIN_ to macth the visual-virtual-reality FR_VIS_ map. (c): orientation of the visual-environment frame FR_VIS_, the kinaesthetic-wrist-centered frame FR_KIN_ and the head-centerd frame FR_H_ during a pointing movement in different experimental conditions. The aiming error is due to the instantaneous angular mismatch between the two frames of reference.

Figure 6 shows the orientations of the *kinesthetic-wrist-centered* frame FR_KIN_ and the *visual* frame of reference FR_VIS_, during the unimodal (**V** or **K**), and bimodal (**VKP**, **VK−** and **VK+**) in a single pointing movement. For a better comprehension we will refer to four different directions during each of these pointing tasks:

- **d_kin_** and **d_vis_** which are the *desired* direction of movements in the *kinaesthetic-wrist-centered* (FR_KIN_) and *visual* (FR_VIS_) frame respectively; the former is the direction along which the subject should aim to move his/her wrist to perfectly match and visualize the straight path (the latter) to the target on the screen.
- **a_kin_** and **a_vis_** which are the *actual* directions of movements in the *kinaesthetic-wrist-centered* (due to wrist movements) and *visual* frame, respectively.

From figure 6.a let's start considering the unimodal visual disturbance: in this experimental condition as explained in the methods section, the FR_VIS_ is continuously rotated according to an harmonic oscillation, varying the orientation of the *visual* frame FR_VIS_ with respect to the *kinaesthetic-wrist-centered* frame FR_KIN_, which is held in a neutral anatomical orientation. If we consider the time instant at which the rotational misalignment between the two frames of reference is θvis, in order to point the target (blue circle) using the cursor (red circle), the subject attempts to move in the FR_KIN_ frame along the **a_kin_** direction, instead of **d_kin_** (Figure 6.b) because this direction is parallel, in his allocentric frame FR_H_, with respect to the straight line on the screen corresponding to **d_vis_**. The visualized direction of movement on the screen will be **a_vis_**, which is rotated with respect to the desired direction **d_vis_** by the amount of the actual rotation between the two frames FR_KIN_ and FR_VIS_. The resulting trajectory will present an initial aiming error with the same sign of the angular mismatch between the *visual* and the *kinaesthetic-wrist-centered* frames of reference (Figure 6.c), and the curved path to the target on the screen is due to the feedback correction operated by the subject.

A similar analysis can explain the curved trajectory also in the unimodal kinaesthetic condition, where the instantaneous angular misalignment is now given by a rotation θkin of the FR_KIN_ frame. Regarding the bimodal disturbance when both the FR_VIS_ and FR_KIN_ frames are continuously rotating, the aiming error will be given by the algebraic sum of the instantaneous angular misalignment of the frames (θvis-θkin). When both reference frames rotate, while maintaining zero angular mismatch (**VK+** where θvis-θkin = 0), the aiming error is almost null or comparable with the one during the unperturbed condition (**F**); this is due to the fact that the wrist device, rotating synchronously with the virtual reality, makes the visual frame FR_VIS_ and the kinesthetic frame FR_KIN_ to be coincident: in this condition the **d_kin_** and **d_vis_** directions are coincident as well as **a_kin_** and **a_vis_** and the resulting movements will be closer to the straight line towards the target, typical of the **F** condition.

Therefore, we suggest that in normal conditions vision and proprioception share the same allocentric reference frame and this common frame is used in order to guide pointing movements also when a common perturbation is applied to both sensory channels. In other words, it appears that in the bimodal condition (**VK+**), the perturbation applied to one channel tends to compensate the effect of the perturbation applied to the other channel if the angular information is congruent, allowing the central nervous system to decrease the computational burden associated to the visuomotor transformation. Moreover, this kind of effect does not require a long training as in prism adaptation but is virtually instantaneous, suggesting the existence of a built-in brain machinery for integrating and dynamically recalibrating visual and proprioceptive information.

## Discussion

Wrist pointing is a precision task that requires careful sensorimotor coordination, using of the visual and proprioceptive channels in a synergic way. The subjects were asked to perform a pointing task towards a visual target in dynamically perturbed visuo-manual distortion environment using multisensory integration by means of a kinesthetic cue; the visual and proprioceptive spaces were disrupted by combining harmonic inputs to the reference frames of the visuomotor transformation. The main purpose was to understand the actual spatio-temporal relation between disturbance features and movement performance. During a whole arm reaching action towards a stationary target the computation of motor error is simply evaluated as the difference between the current and desired position in Cartesian space [41]–[44]; on the contrary, in wrist pointing it is not a trivial problem to evaluate how accurately the hand is pointing to the designated target unless the subject can visualize the projection of his aiming direction on a plane of the object' frame. The task is computationally complex in dynamic conditions, when the relationship between the visual and proprioceptive frames of reference changes over time. In the ***V*** condition, which is similar to the well-studied prism-adaptation paradigm, when a target appears in the context of a rotated visual scene the user should rotate the scene back to the standard orientation, coherent with the proprioceptive frame of reference, in order to generate errorless pointing commands. On the contrary, the subjects produce systematic aiming errors which are compatible with the inability to carry out such rotation. It is remarkable that this effect occurs in spite of the fact that the sinusoidal rotation pattern of the visual scene is perfectly predictable: thus the predictability of the disturbance does not imply the ability to use such prediction in the computation of the motor commands that compensate the angular mismatch between the vision and action frames. Figure 5 clearly shows that this error is accounted for by the misalignment (θvis-θkin) between the visual (FR_VIS_) and proprioceptive (FR_KIN_) frames of reference at the moment of presentation of the target. It is quite likely that, with a sufficiently long training time, this inability can be eliminated as happens for prism adaptation: this is also suggested by the result of fig. 4 that shows a small but measurable performance change between the initial and final part of each experimental session. However, we can say that such adaptation time is much longer than the duration of the experimental protocol investigated in this study, suggesting a process of building an internal model for the compensation of the disturbance.

Rather less predictable is the result of condition ***K***, when the visual scene remains fixed with respect to the egocentric reference (θvis = 0). The wrist frame rotates but this rotation does not affect the two degrees of freedom (flexion/extension and abduction/adduction) that are instrumental for the generation of the pointing trajectory. Thus it could be sufficient for the subject to ignore the proprioceptive signal that codes prono/supination and use the visual information for driving directly the two motor commands. But this is not what people do, at least in an initial training phase. They behave in the same manner of the *V* condition, generating aiming errors as a function of the visuo-proprioceptive misalignement θvis-θkin, with the difference that in the *K* condition θvis = 0 instead of θkin.

The measured outcome does not change if both angular signals (θvis, θkin) vary over time: the aiming error is accounted for by the global misalignment (θvis-θkin) at the time instant of the target presentation. Moreover, there is no difference between the ***VK-*** and ***VKP*** conditions because in both cases the misalignment can have any value in the range of motion and target activation can occur randomly in such range. If this explanation is correct one should predict a very small aiming error if the global misalignment is null for any time instant: in our experimental setup this happens if both disturbance angles oscillate with zero phase shift (conditions ***VK+***). The experiments showed that this is the case and the effect was very quickly achieved by all the subjects.

In summary, we think that the experimental data are compatible with the conjecture that, in order to perform errorless vision-guided pointing movements in a dynamic visuomotor task, the visual and proprioceptive frames of references (FR_VIS_ and FR_KIN_) must be aligned. In normal conditions, with stationary and unvaried visual scenes, this common frame is coincident with the egocentric or body-centered frame FR_H_. In the dynamic conditions described in this paper the common frame of reference becomes allocentric and task-dependent. When the two frames of reference are entrained (***VK+***, θvis = θkin), an invariant feature is established in the relationship between the visual and the kinaesthetic inflows and the experiments suggest that the subjects are quick to detect it. In this way a visually perceived target is automatically mapped into the appropriate motor coordinates without any need to compensate the visual rotation of the scene because the two reference frames are coincident. In other words, the adaptation is almost immediate because there is no need to build an internal model for compensating the visual rotation of the scene and the required visuomotor transformation is essentially an identity mapping.

We suggest that this finding can have practical application in a number of remote control applications in which the visual scene of the workspace and the operating tool is fed back to the operator rotated with uncontrolled dynamic rotations. Another typical application, that may get some benefits, is MIS (Minimally Invasive Surgery) where the disorientation is caused by a mismatch between the line of sight of the surgeon and that of the camera controlled by an assistant; hence the direct view of the instrument is replaced by an indirect view with the results that the mapping between action and perception is dramatically changed.

This effect is best characterized by the highly confusing feeling experienced by the majority of people who manipulates for the first time an instrument under endoscopic condition. Only long training and experience can improve the visuomotor performance. Technical solutions for the compensation of planar misorientation are still in the process of being enhanced and validated [45]–[47]. As such, planar misorientation results in increased navigational difficulties and execution time for laparoscopic surgeons [48].

Controlling the tool in these conditions is very difficult and forces the operator to slow down the movements and perform a number trial and error attempts. Our suggestion is to evaluate in real-time the dynamic rotation of the scene and use this information for generating a synchronized proprioceptive disturbance to the arm/wrist responsible for controlling the remote tool/end-effector.

The interplay between mechanisms of multisensory recalibration and adaptation to novel dynamical environments, possibly with robot-generated assistance patterns, will be addressed in future investigations.

## Supporting Information

Movie S1 (9.14 MB ZIP)

## References

[1] Kohler (1964). The formation and transformation of perceptual world..

[2] Stratton GM (1897). Vision without inversion of retinal image.. Psychol Rev.

[3] Anderson R, Romfh R (1980). Technique in the use of surgical tools..

[4] Cuschieri A, Buess G, Perissat J (1992). Operative Manual endoscopic surgery..

[5] Tending F, Jennings RW, Tharp G, Stark L (1993). Sensing and manipulation problems in endoscopic surgery: experiment analysis and observatio.. Presence.

[6] Congedo M, Lécuyer A (2006). The Influence of Spatial Delocation on Perceptual Integration of Vision and Touch.. Presence.

[7] Groen J, Werkhoven PJ (1998). Visuomotor Adaptation to Virtual Hand Position in Interactive Virtual Environments.. Presence.

[8] Pick HL, Hay JC, Martin R (1969). Adaptation to split-field wedge prism spectacles.. J Exp Psychol.

[9] Van Laer E, Schwartz A, Van Laer J (1970). Adaptation to prismatically displaced vision as a function of degree of displacement and amount of feedback.. Percept Mot Skills.

[10] Kitazawa S, Kohno T, Uka T (1995). Effects of delayed visual information on the rate and amount of prism adaptation in the human.. J Neurosci.

[11] Warren DH, Cleaves WT (1971). Visual-proprioceptive interaction under large amounts of conflict.. J Exp Psychol.

[12] Warren DH, Schmitt TL (1978). On the plasticity of visual-proprioceptive bias effects.. J Exp Psychol Hum Percept Perform.

[13] Warren DH (1979). Spatial localization under conflict conditions: is there a single explanation?. Perception.

[14] DiZio P, Lathan CE, Lackner JR (1993). The role of brachial muscle spindle signals in assignment of visual direction.. J Neurophysiol.

[15] Lackner JR, Levine MS (1979). Changes in apparent body orientation and sensory localization induced by vibration of postural muscles: vibratory myesthetic illusions.. Aviat Space Environ Med.

[16] Vindras P, Viviani P (1998). Frames of reference and control parameters in visuomanual pointing.. J Exp Psychol Hum Percept Perform.

[17] Wigmore V, Tong C, Flanagan JR (2002). Visuomotor rotations of varying size and direction compete for a single internal model in motor working memory.. J Exp Psychol Hum Percept Perform.

[18] Brown LE, Rosenbaum DA, Sainburg RL (2003). Limb position drift: implications for control of posture and movement.. J Neurophysiol.

[19] Sainburg RL, Lateiner JE, Latash ML, Bagesteiro LB (2003). Effects of altering initial position on movement direction and extent.. J Neurophyiol.

[20] Cunningham HA, Vardi I (1990). A vector-sum process produces curved aiming paths under rotated visual-motor mappings.. Biol Cybern.

[21] Abeele S, Bock O (2001). Mechanisms for sensorimotor adaptation to rotated visual input.. Exp Brain Res.

[22] Abeele S, Bock O (2001). Sensorimotor adaptation to rotated visual input: different mechanisms for small versus large rotations.. Exp Brain Res.

[23] Krakauer JW, Pine ZM, Ghilardi MF, Ghez C (2000). Learning of visuomotor transformations for vectorial planning of reaching trajectories.. J Neurosci.

[24] Krakauer JW, Ghez C, Ghilardi MF (2005). Adaptation to Visuomotor Transformations: Consolidation, Interference, and Forgetting.. J Neurosci.

[25] Krakauer JW (2008). Motor Learning and Consolidation: The Case of Visuomotor Rotation. Advances in Experimental Medicine and Biology.. Progress in Motor Control.

[26] Ghilardi MF, Gordon J, Ghez C (1995). Learning a visuomotor transformation in a local area of work space produces directional biases in other areas.. J Neurophysiol.

[27] Wolpert DM, Ghahramani Z, Jordan MI (1995). An internal model for sensorimotor integration.. Science.

[28] Wolpert DM, Ghahramani Z, Jordan MI (1995). Are arm trajectories planned in kinematic or dynamic coordinates? An adaptation study.. Exp Brain Res.

[29] Bernotat R (1970). Operation functions in vehicle control.. Ergonomics.

[30] Kim WS, Ellis SR, Tyler ME, Hannaford B, Stark LW (1987). Quantitative Evaluation of Perspective and Stereoscopic Displays in Three-Axis Manual Tracking Tasks..

[31] Cunningham HA (1989). Aiming error under transformed spatial mappings suggests a structure for visual-motor maps.. J Exp Psychol Hum Percept Perform.

[32] Pennel I, Coello Y, Orliaguet JP (2002). Frame of reference and adaptation to directional bias in a video-controlled reaching task.. Ergonomics.

[33] Pennel I, Coello Y, Orliaguet JP (2003). Visuokinaesthetic realignment in a video-controlled reaching task.. J Mot Behav.

[34] Bock O, Schmitz G, Grigorova V (2008). Transfer of adaptation between ocular saccades and arm movements.. Human Mov Sci.

[35] Girgenrath M, Bock O, Seitz RJ (2008). An fMRI study of brain activation in a visual adaptation task: activation limited to sensory guidance.. Exp Brain Res.

[36] Baugh LA, Marotta JJ (2009). When What's Left Is Right: Visuomotor transformations in an Aged Population.. PLoS ONE.

[37] Kakei S, Hoffman DS, Strick PL (1999). Muscle and movement representation in the primary motor cortex.. Science.

[38] Kakei S, Hoffman DS, Strick PL (2001). Direction of action is represented in the ventral premotor cortex.. Nature Neurosci.

[39] Kakei S, Hoffman DS, Strick PL (2003). Sensorimotor transformations in cortical motor areas.. Neuroscience Research.

[40] Shadmehr R, Wise SP (2005). The Computational Neurobiology of Reaching and Pointing: A Foundation for Motor Learning Cambridge, MA, MIT Press..

[41] Soechting JF, Flanders M (1989). Sensorimotor representations for pointing to targets in three-dimensional space.. J Neurophysiol.

[42] Soechting JF, Flanders M (1989). Errors in pointing are due to approximations in sensorimotor transformations.. J Neurophysiol.

[43] Soechting JF, Flanders M (1991). Arm movements in three-dimensional space: computation, theory, and observation.. Exerc Sport Sci Rev.

[44] Soechting JF, Flanders M (1992). Moving in three-dimensional space: frames of reference, vectors, and coordinate systems.. Annu Rev Neurosci.

[45] Breedveld P, Wentink M (2002). Eye-hand coordination in laparoscopy - an overview of experiments and supporting aids.. Min Invas Ther & Allied Technol.

[46] DeJong BrianP., Colgate J.Edward, Peshkin. MichaelA. (2004). Improving Teleoperation: Reducing Mental Rotations and Translations..

[47] Ellis SR, Nemire K (1993). A subjective technique for objective calibration of lines of sight in closed virtual environment viewing systems..

[48] Wentink M, Breedveld P, Stassen LPS, Oei IH, Wieringa PA (2002). A clearly visible endoscopic instrument shaft on the monitor facilitates hand-eye coordination.. Surg Endosc.

